# Development and Validation of Multi-Locus GWAS-Based KASP Markers for Maize *Ustilago maydis* Resistance

**DOI:** 10.3390/plants14152315

**Published:** 2025-07-26

**Authors:** Tao Shen, Huawei Gao, Chao Wang, Yunxiao Zheng, Weibin Song, Peng Hou, Liying Zhu, Yongfeng Zhao, Wei Song, Jinjie Guo

**Affiliations:** 1 State Key Laboratory of North China Crop Improvement and Regulation, North China Key Laboratory for Crop Germplasm Resources of Education Ministry, Hebei Sub-Center of National Maize Improvement Center of China, College of Agronomy, Hebei Agricultural University, Baoding 071051, China; 17686838765@163.com (T.S.); gaohw77@163.com (H.G.); zhuliying73@163.com (L.Z.); 13582266897@126.com (Y.Z.); 2 State Key Laboratory of Maize Bio-Breeding, National Maize Improvement Center, Department of Plant Genetics and Breeding, China Agricultural University, Beijing 100107, China; sakbj@126.com (C.W.); zyx406939067@163.com (Y.Z.); songwb@cau.edu.cn (W.S.); 3 Key Laboratory of Crop Physiology and Ecology, Ministry of Agriculture and Rural Affairs, Institute of Crop Sciences, Chinese Academy of Agricultural Sciences, Beijing 100081, China; houpeng@caas.cn; 4 Key Laboratory of Crop Genetics and Breeding of Hebei Province, Institute of Cereal and Oil Crops, Hebei Academy of Agriculture and Forestry Sciences, Shijiazhuang 050031, China

**Keywords:** corn smut, disease index, candidate genes, haplotype combinations, marker-assisted selection

## Abstract

Corn smut, caused by *Ustilago maydis*, significantly threatens maize production. This study evaluated 199 maize inbred lines at the seedling stage under greenhouse conditions for resistance to *U. maydis*, identifying 39 highly resistant lines. A genome-wide association study (GWAS) using the mrMLM model detected 19 significant single-nucleotide polymorphism (SNP) loci. Based on a linkage disequilibrium (LD) decay distance of 260 kb, 226 candidate genes were identified. Utilizing the significant loci chr1_244281660 and chr5_220156746, two kompetitive allele-specific PCR (KASP) markers were successfully developed. A PCR-based sequence-specific oligonucleotide probe hybridization technique applied to the 199 experimental lines and 60 validation lines confirmed polymorphism for both markers, with selection efficiencies of 48.12% and 43.33%, respectively. The tested materials were derived from foundational inbred lines of domestic and foreign origin. Analysis of 39 highly resistant lines showed that the advantageous alleles carrying thymine/cytosine (T/C) predominated at frequencies of 94.87% and 53.84%, respectively. The genotype TTCC conferred high resistance, while CCTT was highly susceptible. The resistance exhibited high heritability and significant gene-by-environment interaction. This work systematically dissects the genetic basis of common smut resistance in maize, identifies favorable alleles, and provides a novel KASP marker-based strategy for developing disease-resistant germplasm.

## 1. Introduction

Maize (*Zea mays* L.), a globally essential food and economic crop, is susceptible to multiple pathogens during its growth and development. The seedling stage, a critical phase in maize development, is particularly vulnerable to biotic stresses that significantly impair the morphological formation and physiological functions of plant organs [1]. Corn smut, caused by the basidiomycete fungus *U. maydis*, exhibits broad-spectrum infectivity, targeting leaves and stems at the seedling stage, as well as tassels and ears during reproductive growth, forming characteristic tumor-like structures at infection sites [2]. On a global scale, the yield of maize is reduced by 2–20% per year because of *U. maydis* [3], with infected kernels showing markedly reduced quality, directly compromising commercial value [4]. Resistance to *U. maydis* in maize is characterized as a typical quantitative trait, with its genetic regulatory network involving synergistic interactions among multiple regulatory resistance loci [5]. This polygenic control mechanism results in low efficiency for stacking disease-resistant alleles, posing a major technical bottleneck in maize disease-resistant breeding. Therefore, resolving the molecular genetic mechanisms underlying maize resistance to *U. maydis* and developing resistant germplasm resources are of strategic significance for ensuring the security of maize production.

The corn smut fungus *U. maydis* serves as a model system for biotrophic pathogens, inducing maize smut disease through a specific infection mechanism. Its pathogenic cycle begins with the pheromone-mediated fusion of compatible mating-type haploid cells on the plant surface, forming dikaryotic hyphae regulated by the heterodimeric transcription factor bE/bW [6]. Following cell cycle arrest, these hyphae differentiate into appressoria to penetrate the plant epidermis—a process reliant on cell wall-degrading enzymes and loosening activities—establishing an intracellular colonization state enveloped by the host plasma membrane. Upon penetration, the cell cycle arrest is lifted, and mitotic growth proceeds via a clamp-like nuclear sorting mechanism, extending along the veins towards nutrient-rich tissues [7]. The key pathogenic stage manifests as the induction of abnormal plant cell proliferation leading to tumor formation, within whose cavities hyphal aggregation, nuclear fusion, and massive sporulation occur. Throughout its lifecycle, *U. maydis* strictly maintains its biotrophic nature, establishing a sustained interactive dependency with the host plant [8].

Plants have evolved a sophisticated innate immune system to defend against pathogenic microbial infection, its core being a dual-layer defense mechanism: Pattern-Triggered Immunity (PTI): This is initiated through the recognition of Pathogen-Associated Molecular Patterns (PAMPs) or Damage-Associated Molecular Patterns (DAMPs) by cell surface-localized Pattern Recognition Receptors (PRRs), constituting the basal defense layer that suppresses pathogen invasion and maintains microbial homeostasis [9,10,11,12]. Effector-Triggered Immunity (ETI): This is triggered by the recognition of pathogen effectors via intracellular Nucleotide-Binding Domain Leucine-Rich Repeat Receptors (NLRs), eliciting a robust immune response that includes the localized hypersensitive response (HR) and systemic acquired resistance (SAR), with the latter providing broad-spectrum and durable resistance [13,14]. Salicylic acid (SA) serves as a core regulatory molecule: it participates in both PTI and ETI and promotes the establishment of SAR [12,15]. Pathogens secrete effectors (such as Cmu1, which competes for SA precursors, and Shy1, which degrades SA) to suppress SA accumulation and enhance pathogenicity [16,17], underscoring the critical role of SA in disease resistance [18]. During *U. maydis* infection, the essential virulence factor Cmu1 is secreted into the host cytoplasm and, by competing for chorismite—a precursor shared with the endogenous SA biosynthesis pathway—limits SA synthesis in the host plant, thereby enhancing the pathogen’s virulence [16]. Furthermore, *U. maydis* secretes an effector, Shy1. Shy1, an SA hydrolase, degrades host SA, further suppressing the plant defense response [17].

In previous studies on disease resistance-related genes, integrated approaches combining genome-wide association studies (GWASs), quantitative trait locus (QTL) mapping, and RNA sequencing (RNA-seq) have been employed to identify loci and candidate genes associated with maize resistance, thereby advancing research on mechanisms of maize disease resistance. In studies related to *U. maydis* in maize, previous researchers in our laboratory utilized GWAS and RNA-seq analyses to identify candidate genes associated with maize resistance to *U. maydis*. Through the integration of GWAS and RNA-seq results, five resistance-related genes were identified as key factors in maize resistance to *U. maydis* [1]. Zou et al. analyzed early infection dynamics using inbred line Ye478, with histological and cytological examinations revealing progressive pathogen invasion into host cells from wound sites within six hours post-inoculation, accompanied by detailed documentation of early proliferation in vivo. Concurrent transcriptome profiling across four infection timepoints demonstrated the central role of hormone signaling pathways and glucometabolic and photosynthetic processes in early defense mechanisms, particularly underscoring the essential contributions of chlorophyll biosynthesis and sugar transport to disease resistance [19]. Nisa et al. identified twenty-six significantly associated SNP loci conferring resistance to maize leaf blight through genome-wide association studies. Genes linked to these loci participate in immune responses through fungal activity suppression, enhanced defense against necrotrophic pathogens, and hormone pathway modulation. Further exploration of 22 candidate genes based on functional annotation suggests a polygenic cooperative disease resistance mechanism, providing a basis for integrated breeding strategies [20]. Wang et al. employed BSA-seq and RNA-seq technologies to construct a six-generation population from resistant Qi319 and susceptible Ye478 varieties. Utilizing a major gene-plus-polygene mixed inheritance model, they elucidated the genetic architecture underlying resistance, demonstrating coordinated involvement of photosynthesis, plant–pathogen interaction, carbon fixation, hormone signaling, and MAPK pathways in common smut resistance regulation, thereby establishing a theoretical foundation for resistance gene cloning [21]. Additionally, Tomkowiak systematically identified 92,614 molecular markers via high-throughput sequencing. Association mapping identified sixty-one markers significantly associated with smut resistance, including those markers within *ATAD3*, *EDM2*, and *CYP97A3* genes with substantial breeding value. This research concurrently developed a heterotic group prediction model, providing novel molecular tools for screening disease-resistant germplasm [22]. Baumgarten et al. utilized a novel approach by employing recombinant self-cross populations to map QTLs that govern the frequency and severity of infections by the fungus *U. maydis* in the leaves, ears, and stems of maize plants. This pioneering study laid the foundation for the molecular marker-assisted breeding of maize plants resistant to *U. maydis* [23]. Collectively, these research advances have significantly deepened the understanding of molecular mechanisms underlying maize resistance to *U. maydis* and established a multifaceted theoretical foundation for developing resistant maize cultivars.

KASP markers, a next-generation genotyping technology based on post-PCR fluorescence detection, are characterized by cost-effectiveness, high throughputs, and environmental stability. These advantages enhance breeding efficiency, shorten breeding cycles, and facilitate large-scale applications [24]. KASP markers have been widely adopted for marker-assisted selection (MAS) in crops such as maize, rice, wheat, soybean, and cotton.

For maize, Ertiro et al. compared KASP markers with sequencing-based genotyping in quality control analyses. Despite minor numerical discrepancies between the two methods, their overall conclusions demonstrated high concordance, thereby validating the reliability of KASP markers [25]. Jagtap et al. identified candidate genes associated with heat tolerance in maize through GWAS analysis and subsequently developed and validated 100 KASP markers [26].

Among other crops, Cheon et al. designed 1,225 KASP markers in rice (*Oryza sativa*), which will facilitate gene mapping and molecular marker-assisted selection in breeding programs for temperate japonica [27]. Wu et al. developed and validated 24 KASP markers for QTLs conferring stripe rust resistance in the common wheat (*Triticum aestivum* L.) cultivar P10057 [28], while Tan et al. established 2 KASP markers for the *wheat streak mosaic virus* (WSMV) resistance gene *Wsm2* [29]. Wang et al. performed a GWAS on salt tolerance during soybean (*Glycine max*) germination, screening three highly expressed genes. Additionally, two KASP markers were successfully designed based on SNPs associated with salt tolerance and applied for genotyping soybean germplasm [30]. Jia et al. conducted a GWAS on drought tolerance at the soybean germination stage, identifying 92 SNPs and nine candidate genes significantly linked to drought resistance. Furthermore, two KASP markers closely associated with drought tolerance were developed [31]. Zhao et al. integrated GWAS, QTL-seq, and RNA-seq analyses of Verticillium wilt resistance in 120 elite cotton (*Gossypium hirsutum*) varieties, developing 10 KASP markers targeting Verticillium wilt resistance-associated loci. Validation of marker polymorphism between biparental populations and 26 cotton lines further elucidated the molecular mechanisms underlying resistance to Verticillium wilt in cotton [32].

At present, methods such as GWAS, QTL, and RNA-seq analyses have been widely used in the research on disease resistance of corn, and multiple key SNP loci and important candidate genes related to the disease resistance of corn have been successfully identified. Based on these loci and genes, researchers have developed several KASP markers for molecular breeding practices. Studies of maize resistance to *U. maydis* infection have identified key SNP loci predominantly located in chromosomal bins 1.04/1.08, 2.04/2.05, 4.05/4.09, 5.04/5.09, and 7.02. Nevertheless, the number of KASP markers developed for these loci or associated candidate genes remains limited. This study utilized 199 maize inbred lines as test materials for seedling inoculation tests to screen for highly resistant germplasm resources based on disease indices, thereby providing high-quality germplasm resources for subsequent resistance breeding. GWAS analysis based on disease indices identified significant loci and candidate genes associated with resistance to *U. maydis* in maize. Integrating resistance-related loci with previous RNA-seq data from our laboratory [1], two KASP markers linked to *U. maydis* resistance were developed and validated. Haplotype analysis further screened high-resistance haplotype combinations, significantly improving the selection rate compared to single-marker approaches. This study provides critical insights into the genetic basis of maize resistance to *U. maydis* and facilitates the identification of candidate genes. Furthermore, the application of KASP markers for screening and characterizing maize *U. maydis* resistance germplasm significantly enhances the development of elite cultivars and trait improvement, providing an invaluable structure to support future research on *U. maydis* resistance mechanisms and molecular breeding strategies.

## 2. Results

### 2.1. Analysis of Seedling U. maydis Resistance in Maize Inbred Lines

In the present study, a total of 199 maize inbred lines were utilized as test materials, with B73 serving as the control material. The inoculation of inbred lines with *U. maydis* SG200 occurred at the V3 stage, which corresponded to the eighth day of planting. The analysis of disease incidence on the eighth day following inoculation revealed that most of the materials exhibited comparable susceptibility characteristics. However, a small proportion of the materials demonstrated resistant phenotypes. The seedling evaluation system for resistance, based on the disease index, showed that 199 maize inbreds included 39 highly resistant varieties, accounting for 19.60% of the total materials; 36 resistant materials, accounting for 18.09% of the total materials; and 58 moderately resistant materials, accounting for 29.14% of the total materials; 41 susceptible materials, accounting for 20.60% of the total materials, and 25 highly susceptible materials, accounting for 12.56% of the total materials, were also included in the study.

**Figure 1:**
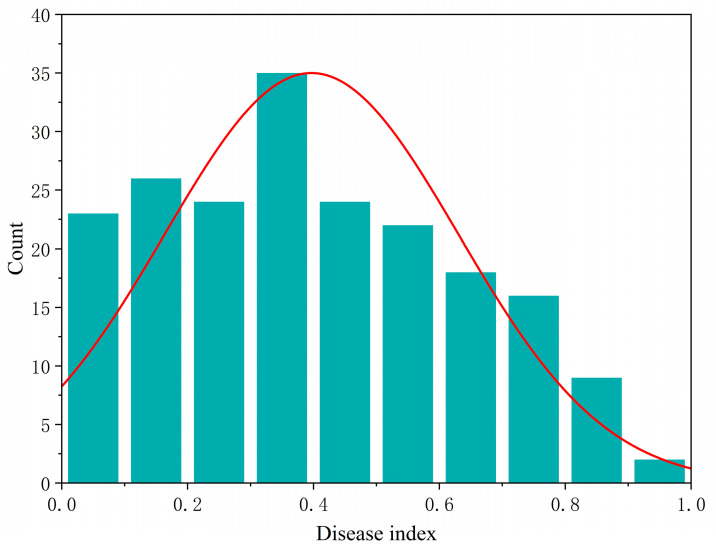
Graph showing the normal distribution of the disease index of 199 maize inbred lines, with the horizontal coordinates indicating the disease index and the vertical coordinates indicating the number of maize inbred lines.

A statistical analysis of 199 maize inbred lines for disease index demonstrated a coefficient of variation of 57.46%; the generalized heritability was 74.94% (Table 1), indicating significant variation in *U. maydis* resistance within this population. Furthermore, the phenotypic data largely conformed to a normal distribution (Figure 1), suggesting that this dataset is appropriate for subsequent statistical genetic analyses.

### 2.2. GWAS Analysis of Maize Inbred Lines’ Disease Indices

**Figure 2:**
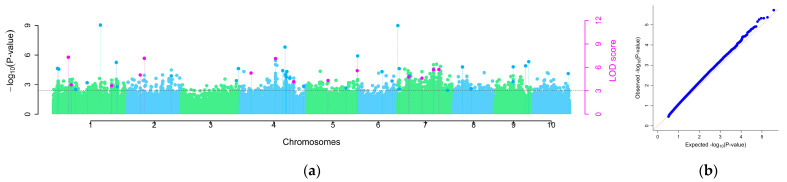
mrMLM software GWAS analysis plots. (**a**) Manhattan plot. Light blue loci are insignificant SNPs, dark blue loci are SNPs detected by one model, and pink loci are SNPs loci detected by two or more methods. (**b**) QQ plot.

After quality control of the genes using PLINK (version 1.9; S. Purcell, C. Chang; Boston, MA, USA) software in this study, 195,108 high-quality SNP markers were obtained. This study employed six models integrated in the mrMLM software package—mrMLM, FASTmrMLM, FASTmrEMMA, ISIS EM-BLASSO, pLARmEB, and pKWmEB—to systematically dissect the genetic architecture of maize *U. maydis* resistance through a multi-locus GWAS. Cross-model integrative analysis identified 88 significant loci (LOD score ≥ 3) distributed across chromosomes 1, 2, 4, 5, 6, 7, 8, 9, and 10 (Figure 2; Table S1). Manhattan and QQ Manhattan plots are integrated plots of the results of six models. In the Manhattan plot, light blue loci represent insignificant SNPs, dark blue loci represent SNPs detected by a single model, and pink loci represents SNPs detected by two or more methods. Model performance varied substantially: the pKWmEB model exhibited the highest detection sensitivity (25 SNPs), followed by pLARmEB (24 SNPs), ISIS EM-BLASSO (18 SNPs), and FASTmrEMMA (15 SNPs), while FASTmrMLM and the baseline mrMLM detected only 6 and 0 SNPs, respectively. Among the 88 SNPs, 19 SNPs loci were detected by two or more models; the LOD scores ranged from 3.01 to 14.44 and the PVE scores ranged from 1.06 to 9.88 (Table S2), predominantly clustered in genomic hotspot regions on chromosomes 1, 2, 4, 5, and 7; multi-model concordance analysis showed locus overlap rates of 52.63% (10/19) between pKWmEB and FASTmrMLM and 36.84% (7/19) between pKWmEB and ISIS EM-BLASSO, suggesting potential major-effect QTLs in these regions. Haplotype analysis further demonstrated that allelic combinations at high-confidence loci were significantly associated with seedling-stage *U. maydis* resistance phenotypes (*p* < 0.001).

### 2.3. Identification of Candidate Genes for Maize U. maydis Resistance

**Figure 3:**
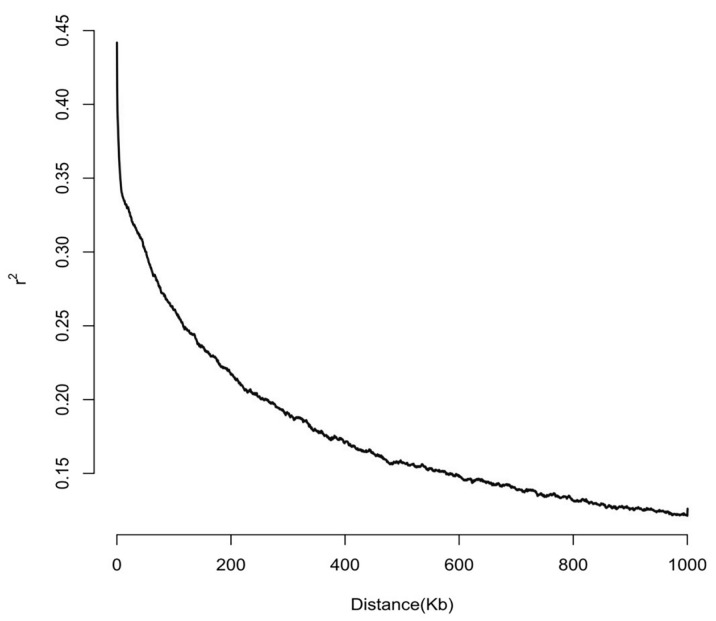
Plot of LD decay for 199 maize inbred lines.

The preceding analyses demonstrated that a total of 88 significant SNP loci were localized by the six mrMLM models, of which 19 were detected by two or more models. In accordance with the LD decay feature, it was established that when r^2^ = 0.20, the average decay distance was 260 Kb (Figure 3). Therefore, the SNP sites were extended upstream and downstream by 260 kilobases each, resulting in a total span of 520 Kb, constructing the search window for candidate genes. Protein-coding genes within these windows were systematically screened using the genome browser tool in the maize GDB database based on B73 RefGen_V4 annotations. The results demonstrated that a total of 671 candidate genes were screened for the 88 significant single-nucleotide polymorphism (SNP) loci, of which 226 candidate genes for the 19 significant SNP loci were detected by multiple models (Tables S3 and S4).

### 2.4. Identification of Important SNP Loci and Candidate Genes

Based on prior RNA-seq studies from our laboratory, three loci significantly associated with resistance to maize *U. maydis* were identified from 19 key SNP sites: chr1_244281660, chr5_220156746, and chr7_88700440. Among these, chr5_220156746 and chr7_88700440 were co-detected by four and five mrMLM models, yielding 39 and 8 candidate genes, respectively. Chr1_244281660 was detected by two models (pLARmEB and pKWmEB), identifying 17 candidate genes. Notably, locus chr2_87571650 was also detected by all five models, revealing one candidate gene (Table S5).

At locus chr1_244281660, two critical candidate genes were determined: *Zm00001d032948*, encoding a fatty acid elongate, and *Zm00001d032946*, encoding chitinase 1. Within the disease-resistant QTL hotspot region at 5_220156746 on chromosome 5, two core candidate genes were identified: *Zm00001d018414*, which encodes Aux/IAA family protein *IAA9*—a key regulator in auxin signaling that mediates transduction by interacting with ARFs (auxin response factors)—and *Zm00001d018421*, which encodes transcription factor *GATA8*, which binds specifically to the *cis-element GATA motif (HGATAR)* through its *zinc finger domain* to modulate immunity-related gene expression. At locus 7_88700440 on chromosome 7, one pivotal candidate gene, Zm00001d020043, was identified, encoding an *ethylene-responsive AP2-EREBP transcription factor* crucial for regulating plant development and responses to diverse biotic/abiotic stresses. At locus 2_87571650 on chromosome 2, candidate gene *Zm00001d004159* was identified, encoding the SNARE-interacting protein *KEULE*. *KEULE* represents a *vital Sec1/Munc18 (SM) family* member in plants, where SM proteins act as key regulators of SNARE complex assembly and membrane fusion—processes essential for plant disease resistance through precise vesicle trafficking (Table 2).

### 2.5. GO and KEGG

**Figure 4:**
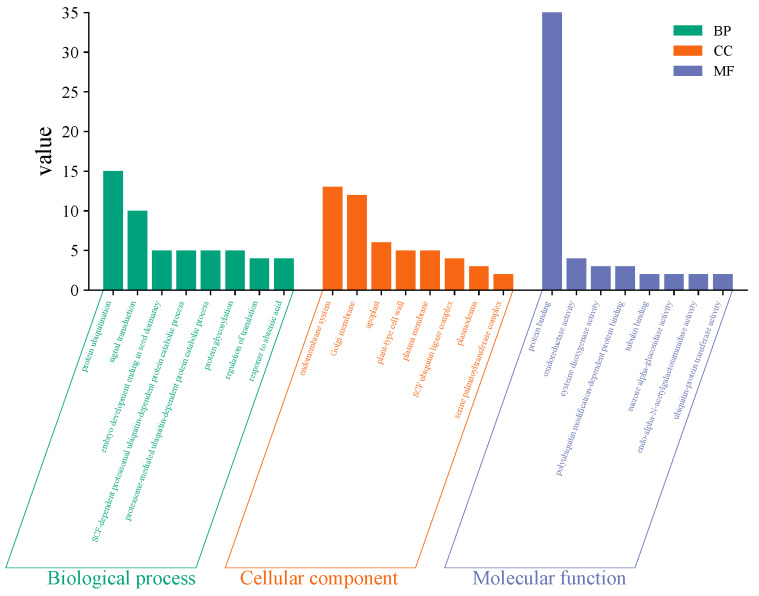
GO plots of candidate genes.

**Figure 5:**
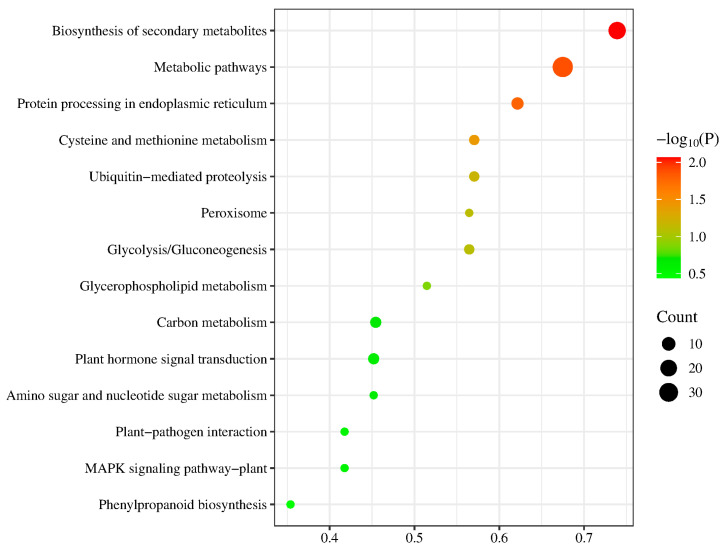
KEGG plots of candidate genes.

To further validate the biological functions of candidate genes, Gene Ontology (GO) and Kyoto Encyclopedia of Genes and Genomes (KEGG) pathway enrichment analyses were performed on 671 candidate genes (Figure 4 and Figure 5). Gene Ontology (GO) is primarily classified into biological processes, cellular components, and molecular functions. Biological processes mainly include protein ubiquitination, signal transduction, and the SCF-dependent proteasomal ubiquitin-dependent protein catabolic process. These processes act as direct executors of immune signaling and pathogen responses or indirectly enhance disease resistance by maintaining cellular homeostasis, regulating gene expression, or improving stress tolerance. Cellular components primarily comprise the endomembrane system, Golgi membrane, and apoplast. These components serve as critical executors and signaling hubs in immune responses, playing decisive roles in defending against pathogen invasion or indirectly influencing defense reaction efficiency through substance synthesis, transport, or metabolic regulation. Molecular functions chiefly encompass protein binding, oxidoreductase activity, and cysteine dioxygenase activity. These functions operate as direct executors of immune signaling and pathogen defense or indirectly impact disease resistance efficiency through metabolic regulation, signal amplification, or structural modification. Among the key loci, the gene *Zm00001d032943* at chr1_244281660 was detected in the biological process of protein ubiquitination, along with the molecular functions of protein binding and oxidoreductase activity. *Zm00001d032944* was detected in the biological process of plant ovule development. These genes may exhibit significant associations with disease resistance. KEGG analysis revealed that these genes predominantly participate in ubiquitin-mediated proteolysis, cysteine and methionine metabolism, glycolysis/gluconeogenesis, carbon metabolism, plant hormone signal transduction, protein processing in the endoplasmic reticulum, biosynthesis of secondary metabolites, and metabolic pathways, with the most enriched pathways being biosynthesis of secondary metabolites and metabolic pathways. Among the four critical loci, genes *Zm00001d032948* and *Zm00001d032950* at chr1_244281660 are involved in both the biosynthesis of secondary metabolites and metabolic pathways.

### 2.6. Haplotype Analysis

**Figure 6:**
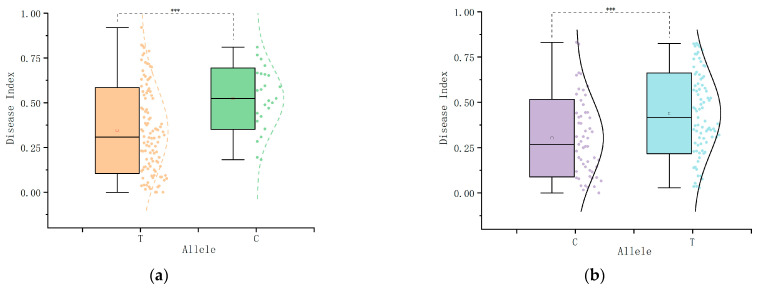
(**a**,**b**) Haplotype box plots of loci 1_244281660 and 5_220156746, respectively, with alleles as the horizontal coordinates and disease indices as the vertical coordinates; *** indicate significant differences at *p* < 0.05, *p* < 0.01, and *p* < 0.001 levels.

The four significant SNP loci identified above were evaluated for KASP marker development, among which two significant loci, chr1_244281660 (T/C) and chr5_220156746 (C/T), were found to be suitable for KASP marker development. To explore the phenotypic effects caused by allelic variations in the two SNPs, haplotype analyses were performed on the significant SNP loci. The results demonstrated significant variations between the different genotypes. The locus 244281660, located on chromosome 1, consisted of a variant of both T/C alleles. The average disease index of the T-containing allele was found to be significantly lower than that of the C-containing allele (Figure 6a), indicating that the T allele functions as a favorable allele for maize *U. maydis* resistance. At locus chr5_220156746 on chromosome 5 (C/T), materials harboring the C allele showed a markedly reduced mean disease index relative to those with the T allele (Figure 6b), demonstrating that the C allele acts as a favorable allele for maize *U. maydis* resistance.

### 2.7. Design Development, Effect Analysis, and Validation of KASP Markers

#### 2.7.1. Design and Development of the KASP Markers

In this study, genomic DNA was extracted from 199 maize inbred germplasms, and two KASP markers, qSB1-KASP and qSB5-KASP, were successfully developed using the above GWAS-significant loci, chr1_244281660 (T/C) and chr5_220156746 (C/T), which were significantly correlated with resistance to *U. maydis* in maize (Table 2). The significant locus chr1_244281660 (T/C) controls genes *Zm00001d032948* and *Zm00001d032946*. *Zm00001d032948* encodes a fatty acid elongate whose enhanced activity increases epidermal wax layer thickness, significantly improving the plant’s physical barrier resistance against *U. maydis*. *Zm00001d032946* encodes chitinase 1, which may recognize and degrade pathogen cell walls during plant defense responses, thereby enhancing disease resistance. The significant locus chr5_220156746 (C/T) regulates genes *Zm00001d018414* and *Zm00001d018421*. *Zm00001d018414* encodes an Aux/IAA family member *IAA9* protein involved in plant growth regulation and disease resistance defense responses, while *Zm00001d018421* encodes a *GATA8* transcription factor that modulates immune-related gene expression. Each marker consists of two forward primers and one reverse primer (Table 3). For qSB1-KASP and qSB5-KASP, the two forward primers were labeled with distinct fluorescent tags, FAM and HEX, respectively.

#### 2.7.2. Utility Analysis of the KASP Markers

**Figure 7:**
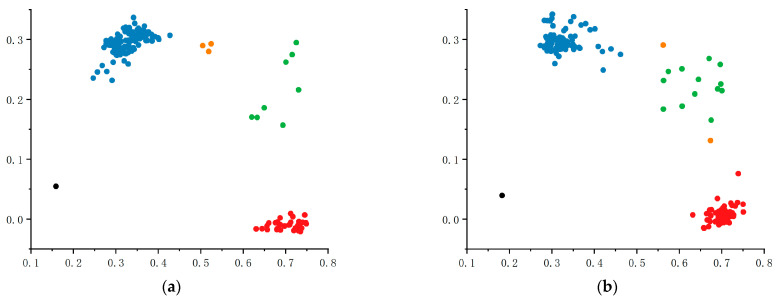
KASP marker genotyping map and haplotype map. (**a**,**b**) Figures show the KASP genotyping plots of chr1_244281660 and chr5_220156746 loci, respectively; in the plots, each point represents 1 inbred line, red indicates the pure genotype CC, blue indicates the pure genotype TT, green indicates heterozygous, orange indicates the failure of the typing material, and the black dots are the blank controls.

The 199 inbred lines were genotyped using markers qSB1-KASP and qSB5-KASP. For qSB1-KASP (T/C), the population was classified into three groups (Figure 7a): 135 TT-genotype materials represented by blue plots near the Y-axis, 56 CC-genotype materials represented by red plots near the X-axis, and 8 heterozygous TC-genotype materials shown as green intermediate plots. This genotyping result demonstrates a DNA polymorphism of this marker in the population. Haplotype analysis revealed that maize carrying the TT genotype exhibited significantly higher maize *U. maydis* resistance values compared to CC-genotype materials, indicating the positive effect of the TT genotype on improving maize *U. maydis* resistance. For qSB5-KASP (T/C), the population was similarly divided into three groups (Figure 7b): 112 TT-genotype materials represented by blue plots near the Y-axis, 72 CC-genotype materials shown as red plots near the X-axis, and 13 heterozygous TC-genotype materials displayed as green intermediate plots, confirming DNA polymorphism of this marker. Haplotype analysis demonstrated that CC-genotype materials displayed significantly higher maize *U. maydis* resistance values compared to TT-genotype counterparts, suggesting the positive role of the CC genotype in enhancing maize *U. maydis* resistance.

#### 2.7.3. Validation of KASP Markers

**Figure 8:**
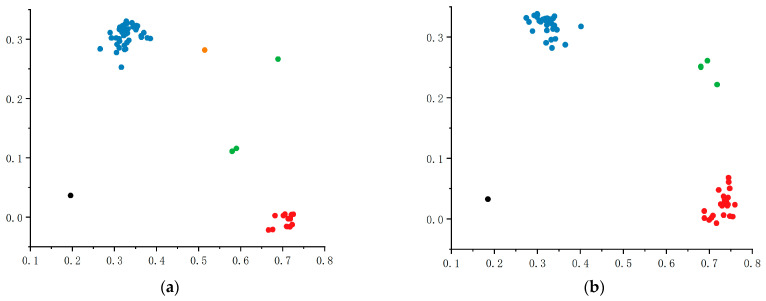
(**a**,**b**) Figures show the KASP genotyping plots of 60 validation materials of chr1_244281660 and chr5_220156746 loci, respectively; in the plots, each point represents 1 self-cross, red indicates pure genotype CC, blue indicates pure genotype TT, green indicates heterozygous, orange indicates typing failure materials, and black dots are blank controls.

**Figure 9:**
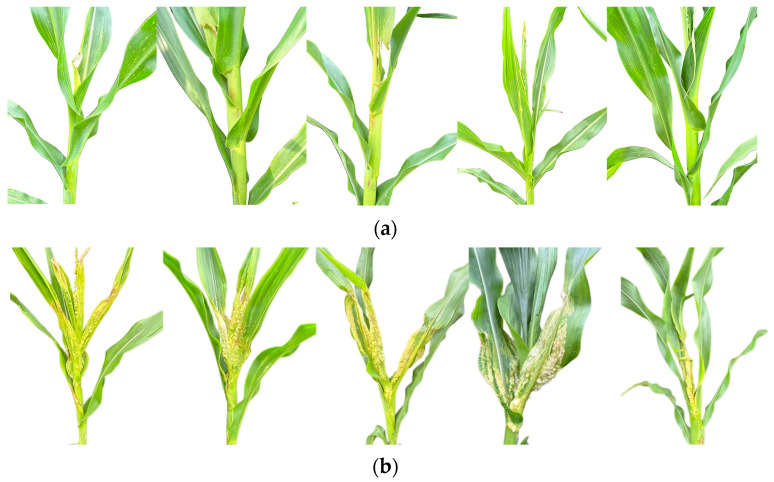
(**a**) The results of the field experiment with five highly resistant inbred lines. (**b**) The results of the field experiment with five highly susceptible inbred lines.

Genotyping of 60 maize inbred lines in the validation population using qSB1-KASP and qSB5-KASP markers revealed successful differentiation of alleles at the significant loci chr1_244281660 and chr5_220156746. For qSB1-KASP (Figure 8a), the population comprised 42 TT-genotype materials by blue plots near the Y-axis, 14 CC-genotype materials by red plots near the X-axis, and 3 heterozygous TC-genotype materials, with the TT allele identified as the favorable allele conferring positive effects on maize *U. maydis* resistance. For qSB5-KASP (Figure 8b), genotyping results showed 24 CC-genotype materials by red plots near the X-axis, 32 TT-genotype materials by blue plots near the Y-axis, and 4 heterozygous TC-genotype materials, with the CC allele serving as the favorable allele associated with enhanced maize *U. maydis* resistance. These results demonstrate the polymorphism of qSB1-KASP and qSB5-KASP markers, confirming their applicability for detecting or validating resistance to *U. maydis* in other maize populations. Subsequently, the five highly resistant inbred lines and five highly susceptible inbred lines jointly identified by the two KASP markers were subjected to field inoculation assays; the results indicated that the five highly resistant inbred lines remained disease-free or exhibited slight discoloration, while the five highly susceptible inbred lines developed severe symptoms, thus demonstrating the accuracy and practical applicability of the markers (Figure 9).

#### 2.7.4. Application of KASP Markers in the Selection of Highly Resistant Materials

Genotyping analysis of 199 maize inbred lines using qSB1-KASP and qSB5-KASP markers (Table 4) revealed that materials carrying the TT genotype at qSB1-KASP exhibited a mean disease index of 0.3720, while those with the CC genotype showed a higher mean disease index of 0.4272. For qSB5-KASP, the mean disease indices were 0.4021 and 0.3776 for TT- and CC-genotype materials, respectively. Materials with disease indices ≤ 0.3 were classified as resistant. Evaluation of the selection rate (defined as the percentage of samples with disease indices ≤0.3 among materials carrying different allelic variants) demonstrated that 48.12% and 43.33% of materials with disease indices ≤ 0.3 were selected, compared to 25.00% and 33.70% for their counterpart genotypes, indicating the functional utility of these KASP markers in selecting *U. maydis*-resistant materials. Allele analysis of 39 highly resistant materials (Table 5) revealed a 94.87% frequency of the favorable T allele at locus chr1_244281660 and a 53.84% frequency of the favorable C allele at locus chr5_220156746. Notably, six varieties exhibiting the highest resistance and harboring two favorable alleles were screened from 32 highly resistant germplasm resources. These findings provide valuable references for breeding high-resistance maize varieties and contribute novel germplasm resources for disease-resistant maize breeding programs.

### 2.8. Effect of Haplotype Combinations Formed Based on KASP Markers for Maize U. maydis Resistance

**Figure 10:**
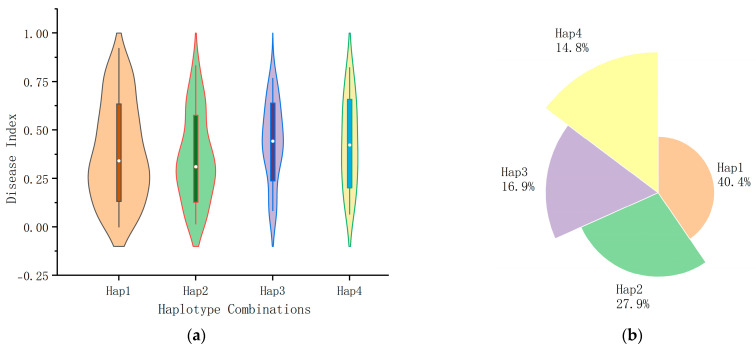
Effects of four haplotype combinations on maize *U. maydis* resistance. (**a**) Graph showing the effect of different haplotype combinations on the disease index, where the vertical coordinate indicates the disease index. (**b**) Graph showing the proportions of the four main haplotype combinations in the 199 material species.

Previous studies have demonstrated that combined markers outperform individual markers in rapidly and accurately targeting related traits [33]. In this study, four haplotype combinations (Table 6) were defined based on the two KASP markers: combination 1 (Hap1-TT), combination 2 (Hap2-TC), combination 3 (Hap3-CT), and combination 4 (Hap4-CC). Haplotype-based grouping of 199 materials revealed that 64 materials belonged to haplotype combination 1, exhibiting a mean disease index of 0.3828 and a selection rate of 43.24%; 41 materials under haplotype combination 2 showed a mean disease index of 0.3423 with a selection rate of 58.82%; 22 materials in haplotype combination 3 had a mean disease index of 0.4380 and a selection rate of 16.67%; and 17 materials in haplotype combination 4 displayed a mean disease index of 0.4302 and a selection rate of 25.92%. Multiple comparative analysis indicated significant effects of different haplotype combinations on resistance to *U. maydis* in maize. Haplotype combination 2 demonstrated the highest resistance, with the lowest mean disease index and the highest selection rate, representing improvements of 10.70% and 15.94% over single-locus selection rates, respectively. These results highlight haplotype combination 2 as the optimal choice for selecting maize *U. maydis*-resistant materials (Table 7, Figure 10).

## 3. Discussion

### 3.1. Maize U. maydis Resistance Identification and Resource Evaluation

The 199 maize inbred lines were evaluated for maize *U. maydis* resistance at the seedling stage using artificial inoculation, leading to the identification of 39 highly resistant materials (Table S6). Allele analysis of these 39 resistant materials (Table 4) revealed a 94.87% frequency of the favorable T allele at locus chr1_244281660 and a 53.84% frequency of the favorable C allele at locus chr5_220156746. Pedigree analysis demonstrated that these resistant materials were widely distributed across maize germplasm groups, including Luda Red Cob, Reid, TangSipingtou, and P groups [1,34]. Previous studies further corroborated these findings: Wang Ting et al. identified five highly resistant materials through injection-based *U. maydis* inoculation using 73 inbred lines, which were clustered into Luda Red Cob and P groups [35]. Similarly, Yan Li et al. screened three disease-resistant materials from 22 maize varieties via seedling-stage artificial inoculation, all belonging to the Tangsipingtou group [36]. These results collectively confirm the presence of resistant materials in the Luda Red Cob, P, and Tangsipingtou groups. This study highlights the diversity of resistance alleles across materials with distinct genetic backgrounds, providing a critical resource foundation and breeding application directions for mining resistance genes against *U. maydis* and disease-resistant germplasm innovation.

### 3.2. GWAS-Significant Loci and Candidate Genes

This study employed the mrMLM software in multi-locus GWAS analysis to screen disease-resistant loci associated with maize *U. maydis* resistance at the seedling stage, identifying a series of SNPs significantly linked to resistance. These loci were predominantly localized on chromosomes 1, 2, 4, 5, and 7. Integrating findings from previous studies in our laboratory, three major loci were ultimately selected, primarily located in the genetic linkage map regions bin1.04/08, bin5.04/09, and bin7.02. Significant progress has been achieved in QTL mapping for maize disease resistance, with multiple research teams elucidating key resistance loci using diverse genetic populations and mapping strategies. Lv et al. identified a Fusarium ear rot (FER)-resistance QTL in the bin1.04 region contributing 10.6% to phenotypic variation via composite interval mapping (CIM) [37]. Wen Jing et al. further revealed cross-population-validated FER-resistance QTL hotspots in regions bin1.04−1.07, bin4.06−4.07, and bin8.05, indicating genetic stability [36]. In corn smut resistance studies, the Qiu team detected a stable and novel resistance QTL in the bin2.04 region using an F2:3 population [38], while Enrico Pè M et al. discovered a Gibberella stalk rot (GSR) resistance QTL in bin5.04, where the candidate gene regulates biotic stress response pathways [39]. In the same chromosomal region (bin5.0), a QTL explaining 13% of phenotypic variation for Fusarium resistance was identified [40]. For gray leaf spot (GLS) resistance, Berger et al. observed QTL enrichment in bin7.02–7.03 through multi-year and multi-environment trials, highlighting this region as a stable multi-environment resistance hotspot [41]. These studies not only clarify the genetic architecture of maize disease resistance but also provide target intervals for molecular marker development. However, some QTLs exhibit environmental interactions or genetic background dependencies, necessitating functional genomics to unravel their molecular mechanisms. Beyond loci consistent with prior research, this study identified resistance SNPs in novel regions: bin1.08 on chromosome 1 and bin4.09 on chromosome 4. These discrepancies may stem from genetic background heterogeneity across studied maize populations, leading to substantial allelic diversity and variation.

This study identified two key candidate genes, *Zm00001d032948* and *Zm00001d032946*, in the association hotspot region of chromosome 1 through joint localization using three models. Previous RNA-seq analyses from our laboratory revealed significant differential expression patterns of these genes in the B73 variety within 48 h post-inoculation with SG200 [1]. *Zm00001d032948* encodes a fatty acid elongate, a critical regulator of plant epidermal wax barrier formation, which determines the chain length and structural arrangement of alkane components in wax crystals by catalyzing the elongation of very-long-chain fatty acids (VLCFAs) [42]. Functional validation demonstrated that enhanced enzyme activity increases epidermal wax layer thickness, significantly improving physical barrier resistance against *U. maydis*, uncovering a novel defense mechanism involving VLCFA biosynthesis in plant innate immunity. *Zm00001d032946* encodes chitinase 1, whose NCBI annotation remains incomplete. However, studies indicate that chitinase 1 plays a vital role in plant defense mechanisms [43,44]. As an enzyme hydrolyzing chitin—a major component of many pathogen cell walls—chitinase 1 may enhance disease resistance by recognizing and degrading pathogen cell walls during plant defense responses.

In the disease resistance QTL hotspot region of chromosome 5, two core candidate genes, *Zm00001d018414* and *Zm00001d018421*, were identified via multi-model joint localization. Previous RNA-seq analyses in the laboratory [1] showed significant DEGs in these locations within 48 h post-inoculation with SG200. *Zm00001d018414* encodes an Aux/IAA family member, *IAA9* protein, a key regulator of auxin signaling that mediates signal transduction by interacting with auxin response factors (ARFs). This study revealed the dual regulatory role of auxin signaling in both plant growth and disease resistance. Mechanistic analysis demonstrated that pathogen infection enhances resistance by suppressing auxin signaling, consistent with cross-species evidence such as downregulated auxin pathways during rice black-streak dwarf virus (RBSDV) infection [45] and improved disease resistance via miR393 overexpression suppressing auxin signaling in Arabidopsis [46], confirming the evolutionarily conserved negative regulatory role of this pathway in plant immunity. *Zm00001d018421* encodes a *GATA8* transcription factor that specifically binds the GATA cis-element (motif: HGATAR) via its *zinc finger domain* to regulate immune-related gene expression. The novel role of GATA family members in mediating disease resistance through SA signaling pathways, e.g., Arabidopsis At*GATA8* regulating seed germination, expands the functional understanding of this transcription factor family beyond developmental regulation [47]. Fourth, at the chr7_88700440 locus on chromosome 7, the candidate gene *Zm00001d020043* was identified using five models from the mrMLM software package. *Zm00001d020043* encodes an ethylene-responsive AP2-EREBP transcription factor, which plays a crucial role in regulating plant growth and responses to diverse biotic/abiotic stresses. The *AP2-EREBP* family, unique to plants, confers tolerance to various biotic stresses. For instance, Arabidopsis *RAP2.2* contributes to resistance against *Botrytis cinerea* and ethylene responses [48], highlighting the multifaceted regulatory roles of AP2-EREBP members in plant development and stress adaptation. At locus chr7_88700440 on chromosome 7, candidate gene *Zm00001d020043* encodes an ethylene-responsive AP2-EREBP transcription factor, which plays pivotal roles in regulating plant growth/development and responses to diverse biotic/abiotic stresses. The AP2-EREBP transcription factor family—a class of plant-specific regulatory proteins—confers tolerance to multiple biotic stresses. For instance, Arabidopsis *RAP2.2* modulates resistance against *Botrytis cinerea* and ethylene responses [49]. AP2-EREBP members execute multi-dimensional regulatory functions in plant development and stress adaptation. Concurrently, locus chr2_87571650 on chromosome 2 represents a newly identified site significantly associated with maize *U. maydis* resistance. Gene *Zm00001d004159* encodes SNARE-interacting protein *KEULE*, an essential *Sec1/Munc18 (SM)* protein family member in plants. SM proteins act as key regulators of SNARE complex assembly and membrane fusion. SNARE complexes mediate vesicle–target membrane fusion, constituting the core mechanism of intracellular vesicle trafficking. This process is indispensable for plant development (e.g., cell division, cell plate formation, cytokinesis, and cell elongation) and environmental signal responses—including pathogen assault. While no direct experimental evidence currently establishes *Zm00001d004159* as a major, well-characterized disease resistance gene in maize, its fundamental cellular functions suggest an indirect yet critical role in disease resistance pathways.

In our previous study, a GWAS conducted using GAPIT and mrMLM software co-localized 136 candidate genes. Integration with transcriptome-derived differentially expressed genes (DEGs) identified five co-regulated genes (*Zm00001d032946*, *Zm00001d032948*, *Zm00001d018414*, *Zm00001d018421*, and *Zm00001d020043*) distributed on chromosomes 1, 5, and 7. In the current investigation, GWAS analysis of disease index data from a new population using mrMLM revealed 226 candidate genes. Cross-referencing these with prior transcriptomic DEGs yielded six co-regulated genes (*Zm00001d032946*, *Zm00001d032948*, *Zm00001d018414*, *Zm00001d018421*, *Zm00001d020043*, and *Zm00001d004159*), with chromosomal locations (1, 2, 5, and 7) largely consistent with previous findings. Notably, we identified a novel significant locus on chromosome 2 (Chr2_87571650; peak LOD = 14.44) encoding a SNARE-interacting protein *KEULE*, with the divergence likely attributable to differences in population structure and sample size [1]. Zou et al. investigated early infection processes of *U. maydis* in maize inbred line Ye478, revealing substantial reprogramming of hormone signaling (JA/SA/ET/ABA), carbohydrate metabolism, and photosynthetic pathways within 0-12 h post-inoculation (hpi). Among these pathways, JA/SA/ET/ABA signaling, glycolysis/gluconeogenesis, and photosystems I/II demonstrated particularly close associations with defense responses. GO enrichment analysis of candidate genes in our current study further corroborates this defense mechanism: response to abscisic acid is functionally involved in ABA signaling pathways regulating seed dormancy and stress defense; proteasome-mediated ubiquitin-dependent protein catabolic process and protein ubiquitination operate through the ubiquitin–proteasome system to precisely modulate hormone signaling (JA/SA/ET/ABA) and defense protein stability; sucrose alpha-glucosidase activity mediates sucrose hydrolysis to influence energy metabolism; and signal transduction integrates hormonal and defense signaling networks (particularly JA/SA/ET/ABA). Concurrently, KEGG analysis of candidate genes in this study detected significant enrichment in pathways including plant–pathogen interaction, plant hormone signal transduction, mitogen-activated protein kinase (MAPK) signaling, glycolysis/gluconeogenesis, phenylpropanoid biosynthesis, endoplasmic reticulum protein processing, and cysteine and methionine metabolism. These findings show strong concordance with the maize defense mechanisms against *U. maydis* reported by Zou et al. [19]. Wang et al. identified four resistance candidate genes on chromosomes 4/6 through BSA-seq and RNA-seq analyses: *Zm00001d052827* encodes a key enzyme in the JA signaling pathway that catalyzes methyl jasmonate biosynthesis; *Zm00001d049111* encodes a cysteine protease inhibitor regulating programmed cell death and fungal invasion responses; *Zm00001d037729* participates in plant fatty acid *α-oxidation* pathways and is associated with stress responses; and *Zm00001d036771* encodes a plant glycoprotein responsive to biotic/abiotic stresses. The KEULE protein discovered in this study, functioning as a key regulator of vesicular trafficking, may participate in this defense network by modulating the transport of defensive compounds [21].

### 3.3. Analysis of KASP Marker Applications

MAS has significantly enhanced crop breeding efficiency by precisely screening target genotypes, with its core principle involving the conversion of functional loci into practical molecular markers. For instance, KASP markers targeting exon 11 (Ex11) and exon 22 (Ex22) of the wheat leaf rust resistance gene *Lr34* enabled rapid identification of durable resistance alleles, substantially shortening breeding cycles for resistant cultivars [50]. Liu Y et al. successfully developed two KASP markers for the recessive *XA5* gene in rice bacterial wilt, which effectively determined target gene homozygosity and heterozygosity across laboratory conditions, validated through breeding segregation populations and core germplasm resources, demonstrating high genotyping accuracy and phenotypic consistency for efficient selection of resistant genotypes, facilitating *XA5* genotyping in molecular breeding programs [48]. Zhou et al. designed four KASP markers for four Fusarium head blight (FHB) resistance-associated genes identified via GWAS analysis, validated in two genetic populations, and provided valuable genetic resources for FHB resistance [51].

In this study, GWAS analysis and prior RNA-seq data led to the selection of five candidate genes and three significant loci. Two KASP markers, qSB1-KASP and qSB5-KASP, were successfully developed based on loci chr1_244281660 (T/C) and chr5_220156746 (T/C). Genotyping of 199 materials revealed that alleles T (qSB1-KASP) and C (qSB5-KASP) conferred positive effects on resistance to *U. maydis* in maize. Among materials carrying these favorable alleles, the resistance selection rate reached 48.12% and 43.33%, significantly exceeding those of other genotypes, confirming the utility of these KASP-SNP markers for high-resistance material selection. Successful genotyping of 60 maize inbred lines further validated the polymorphism and applicability of these markers across diverse populations.

In the present study, four significant haplotype combinations of two molecular markers were analyzed to ascertain their effects on resistance. Analysis of variance (ANOVA) demonstrated that different haplotype combinations had a significant genetic effect on resistance traits to *U. maydis*. Haplotype combination 2 exhibited optimal resistance characteristics, with a mean disease index of 0.3423, lower than other combinations. Its comprehensive selection efficiency of 58.82% surpassed single-locus selection rates by 10.70% and 15.94%, demonstrating the superiority of haplotype-based selection over single-marker strategies. These results indicate that multi-marker haplotype analysis enhances selection accuracy for maize *U. maydis* resistance compared to traditional single-locus approaches. Thus, haplotype combination 2 is recommended as a priority marker combination for maize disease resistance breeding, providing a theoretical foundation for efficient MAS systems. The KASP markers developed here, based on functional genes, combine specificity and universality, offering a general technical pathway for targeted improvement of complex resistance traits in multiple crops.

## 4. Materials and Methods

### 4.1. Experimental Materials

#### 4.1.1. Experimental Maize Inbred Lines

The experimental materials in this study consisted of two parts, both of which were provided by the Hebei Sub-Center of the National Maize Improvement Center of Hebei Agricultural University (longitude 115.447448, latitude 38.827133; Baoding, Hebei Province, China) and contained genetically diverse and widely sourced maize inbred lines from domestic and international breeding practices. Chinese foundational maize inbred lines represent the germplasm resource base adapted to major maize-growing ecoregions in China (tropical, subtropical, and temperate zones) [1,34]. The first part encompassed 199 inbred lines, incorporating the experimental control B73 material, which was predominantly employed for the screening of high-quality resistant material and GWAS analysis. The second part consisted of 60 inbred lines, which were primarily utilized for the validation of KASP markers. All experimental materials were cultivated at the Hebei Sub-Center of the National Maize Improvement Center of Hebei Agricultural University.

#### 4.1.2. Test Strains

*U. maydis* SG200 was generously provided by Academician Regine Kahmann of the Max Planck Institute for Terrestrial Microbiology (Marburg, Hesse, Germany; https://www.mpi-marburg.mpg.de/ (accessed on 12 February 2021)). SG200 (a1 mfa2 bE1/bW2) is a solo pathogenic haploid strain derived from FB1 [52,53]. The fungus was engineered to be able to form infectious mycelia without prior haploid coordination of different genotypes [54], allowing it to infect maize seedlings even under greenhouse conditions [19] and develop tumor structures seven days after inoculation [8,55].

### 4.2. Test Methods

#### 4.2.1. Maize Cultivation and SG200 Culture

Maize Cultivation: The substrate was prepared by thoroughly mixing nutrient soil (Pindstrup, Ryomgaard, Denmark) and vermiculite at a 1:1 volume ratio and was then used to fill 32 well seedling trays. Four surface-sterilized seeds of maize inbred lines were sown per cell at a depth of 1.5 ± 0.2 cm. The trays were placed in an intelligent artificial climate chamber under controlled conditions (Ruihua Instrument, Wuhan, China): diurnal temperature gradient of 25 ± 1 °C (day)/28 ± 1 °C (night), 14 h photoperiod (300 μmol·m^−2^·s^−1^ light intensity)/10 h dark cycle, and relative humidity of 70 ± 5%. A drip irrigation system was employed to replenish deionized water every 48 h, maintaining substrate moisture at 60–70% field capacity. Each maize inbred line was replicated twice in a randomized block design throughout the experiment [1].

Randomization Procedure:

Tray Layout: All inbred lines were assigned to tray positions using a randomized complete block design (RCBD), with two biological replicates per line.

Spatial Randomization: Within each tray, the positions of inbred lines were randomized via a computer-generated random number sequence to minimize edge effects and microenvironmental bias.

Assessment Blinding: Disease phenotyping was conducted by two independent evaluators blinded to the inbred line identities and treatment groups.

SG200 Culture: Primary Culture: Activated SG200 was inoculated onto 90 mm potato dextrose agar (PDA) (Solarbio, Beijing, China) plates and incubated in a constant-temperature incubator (LABOTERY LTI-100; Shanghai, China). The incubation was conducted at 28 ± 1 °C for 5–7 days, or until the mycelia fully covered the surface of the medium. Colonies were then transferred to a 4 °C refrigerator for short-term preservation.

Liquid Subculture: The growth area located at the periphery of the primary colony was selected for the experiment, and a sterile punch was utilized to obtain 5 mm diameter clusters. One or two clusters were then inoculated into 15 mL centrifuge tubes containing 5 mL of YEPSL liquid medium (Solarbio), with the procedure conducted under aseptic conditions. Following inoculation, the culture system was placed in a constant-temperature shaker (ZHICHENG ZHWY-50V; Shanghai, China) at 28 ± 1 °C and 180 rpm, and the culture continued for 20 ± 2 h to reach the logarithmic growth stage.

Preparation of Inoculum: A volume of 100–200 µL of the above culture liquid was inoculated into a 250 mL conical flask containing 50 mL of YEPSL liquid medium. Following inoculation, the culture system was placed in the abovementioned constant-temperature shaker at 28 ± 1 °C, 180 rpm, for the second expansion of the culture and cultivated for 10 ± 0.5 h to ensure that the concentration of the bacterial liquid reached the inoculation requirements. Throughout the culture, aseptic operation conditions were strictly maintained, and all media were autoclaved at 121 °C for 20 min (SNAYO MLS-3750; Tokyo, Japan) [1].

#### 4.2.2. SG200 Inoculation, Phenotyping, and Evaluation Criteria

The prepared SG200 suspension was centrifuged at 1500× *g* for 10 min at 4 °C (Sigma Laborzentrifugen 3K30; Osterode am Harz, Germany), and the supernatant was discarded. The pellet was washed 2–3 times with sterile distilled water (Aquapro AWL-6001-H; Chongping, China). Fungal concentration was measured using a UV-Vis spectrophotometer (Thermo Scientific Genesys 150; Waltham, MA, USA), and the suspension was adjusted to OD600 = 1.0–1.5 with sterile ultrapure water. Eight-day-old maize seedlings (V3 stage) were inoculated by injecting 300 ± 100 µL of the adjusted SG200 suspension into the stem 1 cm above the soil surface and 2.5–3 cm below the basal meristem using a sterile 1 mL syringe [1]. Phenotypic evaluation was conducted for 8 days post-inoculation.

**Figure 11:**
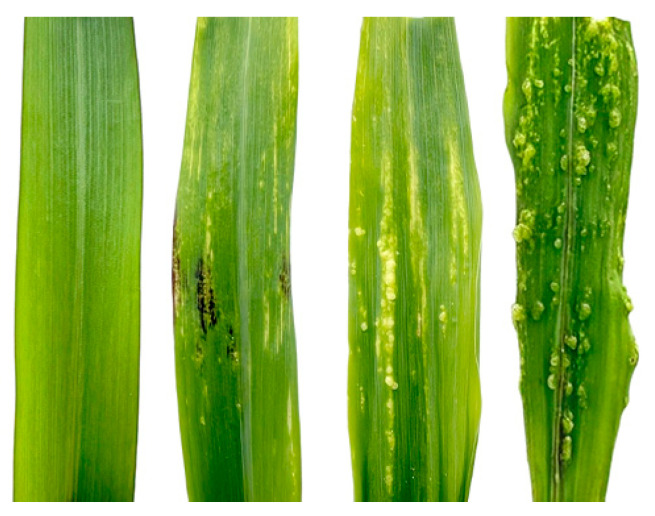
Schematic representation of the different levels of disease after inoculation; from left to right, levels 1 to 4.

Following previous studies [5,56,57], the phenotypic survey was performed on day 8 after inoculation. All the salted seedlings in 32-well seedling trays were removed and categorized into four disease levels according to the degree of symptomatology (Table 8) [1], which was expressed as shown in Figure 11, and the number of salted lines in each disease level was recorded separately.

In this study, seedling resistance criteria for *U. maydis* in maize were determined based on previous research into *U. maydis* resistance in maize (Table 9) [1]. The disease index was calculated based on the seedling disease level using the formula below. The disease index was used for subsequent GWAS analyses [1].

$$Disease\;Index=\frac{\sum \left(a\times b\right)}{N\times K}$$

In the formula, the number of inbred lines at each level is represented by the letter *a*, the representative value of each level is denoted by the letter *b*, the total number of inbred lines investigated is denoted by the letter *N*, and the representative value of the highest level is denoted by the letter *K*.

#### 4.2.3. Extraction of Seedling DNA from Maize Inbred Lines

The DNA sample employed for the development and validation of the KASP markers in this study was extracted using the CTAB method [58]. The extracted DNA samples met the following criteria: a DNA concentration greater than 100 ng/µL, a volume of 100 µL or more, an intact and non-degraded primary band, and a spectrophotometric OD260/280 value of 1.8 or more. In addition, ultrapure water (Aquapro AWL-6001-H Chongping, China) was used as a solvent after DNA extraction to avoid affecting the fluorescence signal interpretation.

#### 4.2.4. GWAS Analysis and Candidate Gene Mining

In single-locus GWAS analysis, markers are tested individually through one-dimensional genome scanning, the primary advantage being the capacity to process large-scale marker data exceeding one million loci. However, this model cannot accurately estimate marker effects, particularly when complex traits are typically controlled by multiple loci, leading to compromised model accuracy. To control the false-positive rate (FPR), multiple testing correction of significance *p*-values is generally required. Although Bonferroni correction serves as a commonly employed adjustment method, its overly conservative nature risks overlooking genuine quantitative trait loci. In contrast, multi-locus models provide a more effective alternative strategy for GWASs. These multi-locus GWAS approaches simultaneously evaluate the genetic effects of all markers via multi-dimensional genome scanning. A notable advantage of these methods is their inherent elimination of the need for Bonferroni correction due to their multi-locus nature. Compared to conventional single-locus GWAS models, multi-locus genome-wide association models have gained widespread recognition in genetics due to their significantly enhanced statistical power and reduced false-positive rates, thereby offering greater alignment with the underlying genetic architecture of plants and animals [59,60,61,62].

In this study, genotyping was performed using the Illumina HiSeq 2000 sequencing platform, yielding 4,644,702 high-quality SNPs [34]. SNP markers were quality-controlled by PLNK software to filter out SNP loci with minimal alleles ≤5% and gene deletions ≥20%. Consequently, 195,108 SNP markers were retained for subsequent GWAS analysis. Six advanced association models in the mrMLM software were applied, including mrMLM, FASTmrMLM, FASTmrEMMA, ISIS EM-BLASSO, pLARmEB, and pKWmEB, with parameters including software default values, a logarithm of ratio (LOD) threshold of ≥3 [63,64], and a two-stage algorithm used for all six models. In the initial phase, the entire genome was subjected to a comprehensive scan utilizing a single-site GWAS approach, and potential QTNs were identified under a less stringent significance threshold. In the second stage, these pre-selected QTNs were subjected to further examination by multi-locus GWAS models, with the objective of identifying authentic QTNs [65,66,67,68,69]. The mrMLM software package provides a solution to the problem of cofactor selection when multiple markers are present in a multi-locus GWAS location, thereby enhancing the accuracy and efficiency of GWAS analysis.

Linkage disequilibrium (LD) constitutes the fundamental theoretical framework of genome-wide association analysis, with its attenuation characteristics exerting a direct influence on the selection of marker densities and the statistical efficacy of association analysis. In the present study, the r^2^ values of SNP loci were utilized to quantify the LD strength. The following values were employed to quantify the strength of LD: r^2^ = 0 characterizes complete linkage equilibrium between loci, while r^2^ = 1 indicates complete LD. The r^2^ values at the critical threshold of 0.2 are of biological significance, suggesting that SNP pairs may be co-localized within the effect compartments of the same quantitative trait loci when r^2^ < 0.2 [70]. This property has been widely employed in the preliminary screening of candidate genes. Following the identification of significant loci using six advanced models, candidate genes related to maize *U. maydis* resistance were identified and screened by maize B73RefGen_V4 and maize GDB (https://www.maizegdb.org/ (accessed on 6 April 2025)) based on LD decay distance.

#### 4.2.5. Haplotype Analysis

Haplotype analysis was performed on significantly correlated locations obtained from GWAS analysis using Haploview 4.2 software [71]. Haploview software generated blocks based on the confidence intervals described [72].

#### 4.2.6. KASP Marker Primer Design and Genotyping

In the present study, GWAS analysis was conducted using the disease index to identify significant SNP loci that were detected by two or more models and were strongly associated with the target traits. Based on the identified SNP loci, the sequences of 100 bp upstream and downstream of the loci were extracted from the maize GDB. The primer design was conducted using the maize GDB platform [73]. The KASP primers comprised three sequences, including two forward primers (FAM and HEX) and one reverse universal primer. The KASP primers featured fluorescent motif connector sequences at the 5′ end, with the FAM and HEX sequences serving as the fluorescent motif connectors. The synthesis of the KASP primers was undertaken by Zhongyu Jinbiao Mark (Beijing, China) Biotechnology Co.

The KASP reaction and subsequent fluorescence detection were carried out on the genotyping detection platform of Zhongyu Golden Marker (Beijing, China) Biotechnology Co. The polymerase chain reaction (PCR) was performed using KASP V4.0 2× Master Mix (LGC KBS-1016-003, Hoddesdon UK), and the amplified deoxyribonucleic acid (DNA) was analyzed using a quantitative real-time polymerase chain reaction (PCR) performed using an ABI QuantStudio 5 system (Applied Biosystems, Foster City, CA, USA) [31]. The PCR reaction mixture (Table 10) and cycling conditions (Table 11) were as follows. The resulting typing results were then subjected to further analysis.

#### 4.2.7. Statistical Analysis of Data

In this study, Microsoft Excel 2022 [74] was utilized for the collection and processing of data, while IBM SPSS Statistics software 26 [75] was employed for descriptive statistical analysis. PLINK software [1] was used for gene quality control, R-4.3.3 [76] for GWAS analysis, and SNP viewer [77] software for the viewing of KASP marker typing results, while GO and KEGG analyses were performed using AVID and SRplot [78] and Origin2024 [79] was used for graphing.

## 5. Conclusions

GWAS analysis identified 88 significant SNPs, with 19 loci detected by two or more models and 225 candidate genes associated with maize *U. maydis* resistance. The significant loci chr1_244281660, chr2_87571650, chr5_220156746, and chr7_88700440 yielded six critical genes—*Zm00001d032946*, *Zm00001d032948*, *Zm00001d004159*, *Zm00001d018414*, *Zm00001d018421*, and *Zm00001d020043*—associated with physical disease resistance, growth and development, and immune-related gene expressions, and they were successfully converted into KASP markers qSB1-KASP and qSB5-KASP. Genotyping results showed selection rates of 48.12% and 43.33% for materials carrying the advantageous alleles T and C, respectively. Haplotype combination analysis revealed that combination 2 exhibited a disease index of 0.3423 and a selection rate of 58.82%, representing increases of 10.70% and 15.94% compared to single-marker selection rates, confirming the efficacy of multi-marker selection. These results provide technical support for establishing a molecular breeding framework for maize disease resistance. In the present study, 199 maize inbred lines were identified for seedling *U. maydis* resistance, and 39 highly resistant materials were screened out, thereby providing high-quality germplasm resources for subsequent resistance breeding. This study systematically elucidated the genetic basis of maize *U. maydis* resistance, identified advantageous allelic resources, and developed a KASP marker system that offers novel strategies for creating disease-resistant germplasm. It lays the groundwork for future research integrating functional genomics to dissect QTL–environment interactions and enables precise utilization of resistance genes.

## Figures and Tables

**Table 1:** Descriptive statistical analysis.

Max	Min	Mean	SD	Variance	Skewness	Kurtosis	CV%	G × E	H^2^%
0.9207	0.0167	0.3991	0.2294	0.0530	0.1870	−0.9550	57.46	0.0016	74.94

Max, maximum; Min, minimum; SD, standard deviation; CV, coefficient of variation; G × E, gene-environment interaction; H^2^%, heritability.

**Table 2:** Candidate genes and their functional annotations.

Significant SNP Locus	LOD Score	Model	Genome	Functional Notes
1_244281660	8.57 6.43	FASTmrMLM pKWmEB	*Zm00001d032946*	Chitinase 1
			*Zm00001d032948*	fatty acid elongase
2_87571650	4.70 3.42 3.85 14.44	FASTmrMLM FASTmrEMMA pLARmEB pKWmEB ISIS E-BLASSO	*Zm00001d004159*	SNARE-interacting protein *KEULE*
5_220156746	5.95 3.02 6.87 3.43	FASTmrMLM FASTmrEMMA pLARmEB pKWmEB	*Zm00001d018414*	*IAA9*—auxin-responsive Aux/IAA family member
			*Zm00001d018421*	*GATA* transcription factor 8
7_88700440	11.48 4.50 13.11 4.60	FASTmrMLM FASTmrEMMA pKWmEB ISIS EM-BLASSO	*Zm00001d020043*	*AP2-EREBP* transcription factor

**Table 3:** Sequence information of qSB1-KASP and qSB5-KASP markers.

Marker Name	Primer Name	Primer Sequence, 5′-3′
qSB1-KASP	1-A	GAAGGTGACC AAGTTCATGC TGACATCTGT CTACGACTAG AGCG
	1-B	GAAGGTCGGA GTCAACGGAT TGACATCTGT CTACGACTAG AGCA
	1-C	TGATCGAACG ATGAGCCCGA TCATA
qSB5-KASP	5-A	GAAGGTGACC AAGTTCATGC TCAGCCCATA CCAAGTGGCT AG
	5-B	GAAGGTCGGA GTCAACGGAT TGCAGCCCAT ACCAAGTGGC TAA
	5-C	GAGGTGAGAG ATACCACAGT CCTAT

A and B denote forward primer representations, and C denotes reverse universal primer.

**Table 4:** Effect of two KASP markers on maize *U. maydis* resistance.

Marker Name	Alleles (Number of Samples)	Mean Value of Disease Index	Selection Rate (Sample Size)	*p*-Value
qSB1-KASP	T (133)	0.3720	48.12% (64/133)	*p* < 0.05
	C (45)	0.4272	26.66% (12/45)	
qSB5-KASP	T (92)	0.4021	33.70% (31/92)	*p* < 0.05
	C (90)	0.3776	43.33% (39/90)	

**Table 5:** Percentages of favorable alleles in highly resistant varieties.

SNP Significant Loci	Percentages of Favorable Alleles	DM07	18	W668	Lo1125	Jingnuo2	DH65232
1_244281660 (T/C)	94.87% (37/39)	T	T	T	T	T	T
5_220156746 (C/T)	53.84% (21/39)	C	C	C	C	C	C

**Table 6:** Haplotype combinations consisting of 2 KASP markers.

Marker Name	Hap1	Hap2	Hap3	Hap4
qSB1-KASP	T	T	C	C
qSB5-KASP	T	C	T	C

**Table 7:** The influence of different haplotype combinations on resistance.

Haplotype Combinations (Sample Size)	Average Disease Index	Selection Rate (Sample Size)	*p*-Value
Hap1 (74)	0.3828	43.24% (32)	*p* < 0.05
Hap2 (51)	0.3423	58.82% (30)	
Hap3 (32)	0.4380	16.67% (3)	
Hap4 (27)	0.4302	25.92% (7)	

**Table 8:** Performance symptoms of disease levels at seedling stage.

Level of Disease	Performance Symptoms
1	No infection symptoms are observed in the plants
2	Minimal infection manifests as chromatic alterations on inoculated leaves
3	Moderate infection produces pathognomonic rice-grain-sized tumors on foliar and sheath tissues
4	Severe infections cause pathological distortion of leaves, stems, and basal culms, accompanied by enlarged verrucous proliferations

**Table 9:** Criteria for evaluating maize *U. maydis* resistance at seedling stage.

Disease Index	Capability to Resist	Abbreviated
0–15.0	Highly resistant	HR
15.1–30.0	Resistant	R
30.1–50.0	Moderately resistant	MR
50.1–70.0	Susceptible	S
70.1–100.0	Highly susceptible	HS

**Table 10:** PCR reaction system for KASP typing detection.

PCR Reaction Components	System Usage (µL)
DNA	1.5
2× Master mix	0.75
KASP marker primer	0.0417
H_2_O	0.75
Total volume	3

**Table 11:** PCR reaction procedures.

Steps	Effect	Temperature (°C)	Time	Number of Cycles
1	Denaturation	94	15 min	1
2	Denaturation	94	20S	10
	Annealing/Extension	61–55	60S	
3	Denaturation	94	20S	26
	Annealing/Extension	55	60S	

## Data Availability

The raw data supporting the conclusions of this article will be made available by the authors on request.

## Supplementary Materials

The following supporting information can be downloaded at https://www.mdpi.com/article/10.3390/plants14152315/s1: Table S1: Significant association sites detected by six models in mrMLM software; Table S2: Significantly associated loci detected by two or more models in mrMLM software; Table S3: Candidate genes for 88 SNP loci detected by mrMLM; Table S4: Candidate genes and functional annotation of 19 important SNPs identified by mrMLM analysis; Table S5: Candidate genes and functional annotation of 4 important SNPs identified by mrMLM analysis. Table S6: Thirty-nine highly resistant maize inbred lines.
